# The nature of beliefs and believing

**DOI:** 10.3389/fpsyg.2022.981925

**Published:** 2022-07-29

**Authors:** Mahault Albarracin, Riddhi J. Pitliya

**Affiliations:** ^1^VERSES Research Lab, Los Angeles, CA, United States; ^2^Département d'informatique, Université du Québec à Montréal, Montréal (Québec), QC, Canada; ^3^Department of Experimental Psychology, University of Oxford, Oxford, United Kingdom

**Keywords:** active inference, beliefs (non-religious), epistemic (belief) changes, socio-political, psychopathology

## The nature of beliefs

Conceptualizations of beliefs differ according to the school of thought considered; here, we take the view from cognitive science.

In cognitive science, beliefs are propositional attitudes, where the world is depicted as being in some state or another (Schwitzgebel, 2021). Beliefs have two main properties: some representational content and assumed veracity (Stephens and Graham, 2004). Beliefs entail specific representational content, which portrays causes of sensations (agency, events, and objects) as being a specific way (Rimell, 2021). So understood, they are undoubtedly a central part of cognition, dictating our perceptions, behavior, and executive functions. Beliefs do not need to be conscious or linguistically articulated, and indeed, the majority of beliefs can be construed as subpersonal; i.e., remain unconscious (Majeed, 2022). Rational agents generally view beliefs as having a truth value, and update their beliefs in light of new evidence. The term “belief” is also used to denote a more deflationary sense, where what is at stake is merely a probability density over some support; where we call a belief a probabilistic assessment of how plausible some state of affairs is (Smets, 2005). On this probabilistic reading, beliefs acquire the attribute of uncertainty—or its complement precision.

Beliefs provide the foundation that allows agents to understand—or at least make sense of—the world and act within it: they provide agents with a consistent and coherent representation of their world, which they can then use to make inferences about the causal structure of the world and their place within it (Churchland and Churchland, 2013). This scaffolding of beliefs helps [human] agents appraise the environment, explain new observations, construct shared perspectives on the world, and engage in goal-directed behavior.

Beliefs also help us experience the world temporally, as they can represent the state of the world in the past and allow us to anticipate its state in the future; this is especially important when holding beliefs about the consequences of action—a prerequisite for planning and a sense of agency (Shipp et al., 2009).

## Active inference

**Figure 1:**
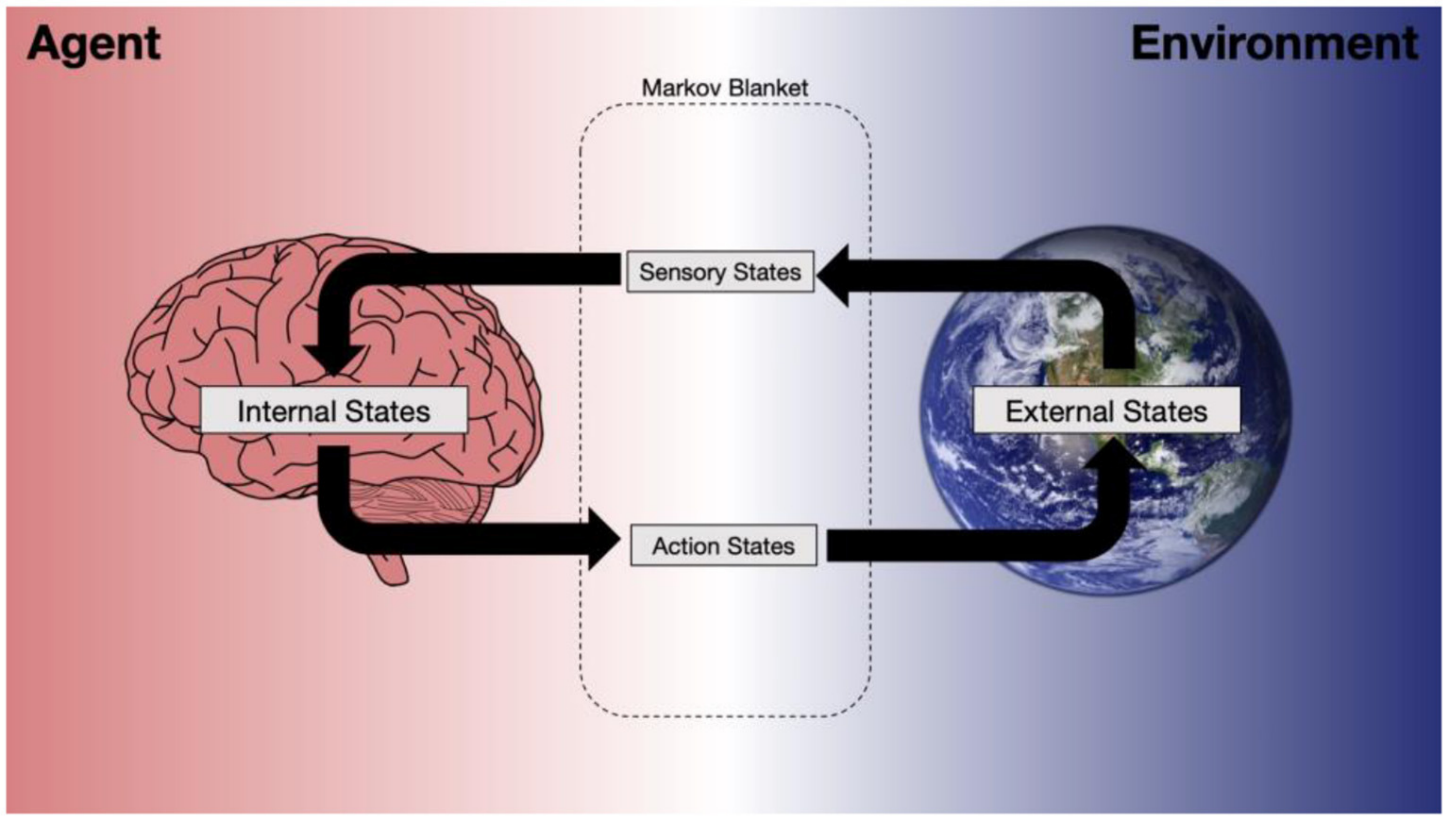
An illustration of how a model of the external world is formed, resulting in beliefs about the true states of the world and belief updating, encoded by internal states.

Active inference is a formal description of self-organization derived from the variational free energy principle, and provides a mechanistic account of belief-guided action (Friston, 2010; Friston et al., 2017). In particular, it is used to model and simulate how beliefs about the states of the world are formed and updated. It proposes to naturalize belief in the first sense by formalizing them as beliefs in the second sense (Friston et al., 2015). In this context, the semantic content of a belief is equated with the support of its probability density (i.e., the external or worldly states over which it is defined). Figure 1 illustrates the dynamic relation between (internal, external, and blanket) states.

### Belief formation

Active inference rests on a particular partition of a system (i.e., into particles), where we distinguish between the states internal and external to a system, as well as the states that constitute the boundary of the system via which it interacts with its environment (Friston, 2010; Friston et al., 2017). The external states are assumed to be hidden from direct observation behind the blanket states. In other words, sensory states allow the agent to access their environment vicariously, by sampling it through action. Internal states encode beliefs about the external world, in the form of sufficient statistics (e.g., an expected value or expectation and the confidence of precision of that belief). The agent thus forms beliefs about the hidden states of the external world that have caused their observations (sensory data). Through this accumulation of observations, agents continuously update their beliefs about external states of affairs, and about the most likely future states—that clearly depend upon action.

### Belief updating

These beliefs are not static. Depending on how well their beliefs enable them to predict the world, agents can update their (Bayesian) beliefs about of the world. This can be read as a Bayesian mechanics in which, agents acuminate and assimilate new evidence, and re-calibrate what they believe to be the cause of their sensory experiences (Ramstead et al., 2022). This process is generally read as minimizing surprise (technically, the negative logarithm of model evidence of observations). This is mathematically the same as maximizing the evidence for a model of the world; sometimes called a world model or generative model—that generates predictions of (observable) sensory states from their (hidden) external causes.

Active states allow the agent to interact with her world, by directly affecting the process generating her sensory data (i.e., the environment), and accordingly update her beliefs about the external world (Of course, new observations may be formed by causes other than the agent herself) (Friston et al., 2016). This enactive aspect of inference now affords the opportunity to plan responses and choose those actions that will minimize surprise and maximize model evidence; sometimes referred to as self-evidencing.

In active inference, policy selection determines the agent's actions. A belief about a current course of action is called a “policy.” In this setting, the agent also forms beliefs about what she is doing, and updates her beliefs about the current course of action (i.e., its policy) based on her other beliefs about the current state of world and the goals that the agent is trying to achieve. The different policies available to an agent are then variations of beliefs about expected future observations, contingent on different courses of action. A policy is selected based on preferences for specific sensory outcomes, by choosing the one that minimizes the divergence between expected and preferred outcomes. This divergence between expected and preferred outcomes is quantified using “expected free energy.” The optimal policy is the one that provides the most evidence for the generative model (or equivalently, that is expected to generate the least expected surprise or uncertainty). Active inference thereby purports to explain the dual aspect of choice behavior; namely preference-seeking and information-seeking behavior, respectively. Both aspects are in the service of avoiding surprises (e.g., non-preferred, outcomes). Technically, expected free energy can be decomposed into expected information gain and expected loss; in the sense of optional Bayesian design and Bayesian decision theory, respectively.

Perception is realized by a process called state estimation (Oliver et al., 2021). Beliefs about the current state of the world are updated, based on priors that encode what the agents believes to be the base rates of occurrence of various states of affairs, the likelihood of current observations being caused by certain states, and the probability that states will transition to other states. All knowledge about past states is implicitly packed into beliefs about the current state: belief updating of this sort satisfies what is known as the Markov property and is the basis of all evidence accumulation and inference. The structures that underlie this kind of belief updating are themselves optimized to maximize model evidence leading to an understanding of learning in terms of optimizing the parameters of a generative model (e.g., learning the weights of a neural network or associative plasticity in neuronal networks).

## Beliefs across scales

The basic mechanics of active inference can be deployed across nested spatial and temporal scales, describing a nested set of agents composed of other agents—e.g., organs composed of cells and communities composed of individuals—thanks to its scale invariant construction (Kirchhoff et al., 2018). Each scale relies on a formulation of self, which is bounded in a way that individuates self from non-self.

These boundaries just are the Markov blankets above: a Markov blanket comprises of the sensory and action states that separates the internal states from external states of a system (see Figure 1). Markov blankets are drawn at the boundary of a system and define the interface where it receives sensory stimulation. Thus, depending on the perspective, an agent can have its own Markov blanket, or can be a part of a higher-order blanketed system, as a component of that system (Kirchhoff et al., 2018).

### Shared beliefs

A Markov blanket can also be drawn over a group of agents. These agents gain an advantage, namely, they can pool their resources, gathering evidence as a group without having to expend the effort necessary to acquire in isolation. The individual agents forming a higher-order agent thereby improve their own predictive abilities and minimize expected free-energy as a group, aligning their beliefs as they share their beliefs about the (co-constructed) world. In the ideal case, agents generate evidence for a shared model of the world. The more similar each agent's model is, the more likely they are to minimize surprise, predict each other and resolve uncertainty, i.e., jointly self-evidencing. Thus, sampling from members of a group that share a similar model itself provides significant informational and evolutionary benefits and allows groups of agents to optimize their beliefs over longer timescales than would otherwise be possible; c.f., cultural eco-niche construction (Bouizegarene et al., 2020).

## Applications

We can use the definition of beliefs as propositions of the true—but unknowable—states of the world, and the formalization of belief formation and updating as proposed by active inference, to understand several socio-political and psychopathological phenomena.

### Socio-political phenomena

When two agents interact, the value of their beliefs is weighed against the beliefs and observations to which the individual agents already have access. It is advantageous for individuals to be part of a group in terms of large-scale coordination, available resources, and computational power. But it is costly to change any single agent's deeply held beliefs. Therefore, individual agents will give more weight to sensory evidence from agents who are believed to share the same generative model or world narrative. The more agents in a group consider some data points as valuable, and as constituting evidence for their beliefs, the more likely it is that the agents in that group will conform to the beliefs of the group. This is a form of epistemic confirmation bias. In extreme cases, agents can then fall into “echo chambers,” in which their beliefs no longer align with any other interpretation of the world, outside that of their own group. We can see this phenomenon at play with misinformation on social media, spreading extremely fast in echo-chambers, and leading to radicalization (Albarracin et al., 2022).

When a group exists long enough and shares a core set of beliefs, it tends to leave traces in the environment that reflect those beliefs. Consider agents walking in a park. As they walk on the grass, they tend to leave footprints, where the grass will be less likely to grow. Agents noticing these traces may be likely to follow a similar path, reinforcing the path in the grass. This recursive process engraves shared beliefs in the structure of the environment, serving as an anchor around which narratives and semantics will be carried through generations of agents in similar groups. As agents continue to produce shared beliefs, they become embedded in a culture and a cultural materiality, which allows them to predict—and navigate—each other and their environment more easily (Ramstead et al., 2020).

### Psychopathology

Active inference is often used in computational psychiatry, to characterize psychiatric conditions as a consequence of atypical beliefs. Strange and untrue beliefs may form based on an individual's experience of the world and the structure of their beliefs, giving rise to psychiatric conditions.

Consider schizophrenia, which has been characterized by a failure of sensory attenuation, with secondary consequences for the dysfunctional acquisition of beliefs about the world and interpersonal interactions (Brown et al., 2013). In the context of sensory exchanges with the world, a failure of sensory attenuation means that the weight of the sensory data (observations) is too high, relative to the prior beliefs about the causes of sensations (Friston et al., 2014). This may explain the inability of schizophrenic patients to infer regular contingencies between stimuli in the world. However, since the prior beliefs are themselves compromised, surprising new data paradoxically improves the detection of new stimuli and contingencies. At the same time, this may also result in such individuals misinterpreting ambiguous information and inferring contingencies that do not exist, resulting in inaccurate beliefs (or delusions). For example, individuals with schizophrenia tend to fail to contextualize the consequences of their actions and thus possess false beliefs about their agency (Jeannerod, 2009). Such individuals may also “jump to conclusions”—i.e., they may require less evidence to form a strong belief (FitzGerald et al., 2015).

If an individual's beliefs cannot be flexibly updated, they may consistently act in a way to align their observations with their beliefs, which would result in dysfunctional behavior. Such individuals would not be able to update their beliefs, even when confronted with new observations that challenge their current beliefs. This is a common symptom observed in individuals with depression, where they tend to not act in their environment even when they do have control, giving rise, over time, to a sense of learned helplessness, and to low motivation and inaction (Grahek et al., 2019).

## Summary

Beliefs are propositions about the true states of the world. Active inference—a process theory based on the free energy principle—describes how an agent forms and updates beliefs. The active inference framework posits that the agent (i) observes the world, (ii) infers the causes of the observations, and (iii) forms beliefs about the external states of the world. The agents then act in the world, prompting new observations, and thereby update their beliefs. These beliefs underwrite how an agent approaches the world, and how they will navigate through it, given possible paths into the future. Modeling how agents update their beliefs is thus central to understanding both micro- and macro-phenomena, such as deviations in beliefs at an individual level, resulting in dysfunctional behavior and the development psychiatric conditions. And, at the group level, allowing us to better understand socio-political dynamics in multi-agent scenarios.

Our commentary is an addition to Credition—An Interdisciplinary Approach to the Nature of Beliefs and Believing. In this edition, Seitz, Angel, Paloutzian and Taves have put together a series of perspectives on believing which furthers our understanding of the ways beliefs play a part in cognition, technology and science, ranging from understanding how beliefs are shaped, to their role in artificial intelligence, to the role of culture in beliefs, and more.

## Author contributions

All authors listed have made a substantial, direct, and intellectual contribution to the work and approved it for publication.

## Funding

This article was funded by RS, via the Volkswagen Foundation, Siemens Healthineers, and the Betz Foundation and the VERSES computational phenomenology lab.

## Conflict of interest

The authors declare that the research was conducted in the absence of any commercial or financial relationships that could be construed as a potential conflict of interest.

## Publisher's note

All claims expressed in this article are solely those of the authors and do not necessarily represent those of their affiliated organizations, or those of the publisher, the editors and the reviewers. Any product that may be evaluated in this article, or claim that may be made by its manufacturer, is not guaranteed or endorsed by the publisher.
